# Repeated cycles of 5-fluorouracil chemotherapy impaired anti-tumor functions of cytotoxic T cells in a CT26 tumor-bearing mouse model

**DOI:** 10.1186/s12865-016-0167-7

**Published:** 2016-09-20

**Authors:** Yanhong Wu, Zhenling Deng, Huiru Wang, Wenbo Ma, Chunxia Zhou, Shuren Zhang

**Affiliations:** 1 Department of Immunology, National Cancer Center/Cancer Hospital, Chinese Academy of Medical Sciences & Peking Union Medical College, Beijing, People’s Republic of China; 2 Department of Blood Transfusion, Anhui Provincial Hospital, Hefei, People’s Republic of China

**Keywords:** Chemotherapy, Immune functions, Cytotoxic T cells, Immunotherapy, Cancer

## Abstract

**Background:**

Recently, the immunostimulatory roles of chemotherapeutics have been increasingly revealed, although bone marrow suppression is still a common toxicity of chemotherapy. While the numbers and ratios of different immune subpopulations are analyzed after chemotherapy, changes to immune status after each cycle of treatment are less studied and remain unclear.

**Results:**

To determine the tumor-specific immune status and functions after different cycles of chemotherapy, we treated CT26 tumor-bearing mice with one to four cycles of 5-fluorouracil (5-FU). Overall survival was not improved when more than one cycle of 5-FU was administered. Here we present data concerning the immune statuses after one and three cycles of chemotherapy. We analyzed the amount of spleen cells from mice treated with one and three cycles of 5-FU as well as assayed their proliferation and cytotoxicity against the CT26 tumor cell line. We found that the absolute numbers of CD8 T-cells and NK cells were not influenced significantly after either one or three cycles of chemotherapy. However, after three cycles of 5-FU, proliferated CD8 T-cells were decreased, and CT26-specific cytotoxicity and IFN-γ secretion of spleen cells were impaired in vitro. After one cycle of 5-FU, there was a greater percentage of tumor infiltrating CD8 T-cells. In addition, more proliferated CD8 T-cells, enhanced tumor-specific cytotoxicity as well as IFN-γ secretion of spleen cells against CT26 in vitro were observed. Given the increased expression of immunosuppressive factors, such as PD-L1 and TGF-β, we assessed the effect of early introduction of immunotherapy in combination with chemotherapy. We found that mice treated with cytokine induced killer cells and PD-L1 monoclonal antibodies after one cycle of 5-FU had a better anti-tumor performance than those treated with chemotherapy or immunotherapy alone.

**Conclusions:**

These data suggest that a single cycle of 5-FU treatment promoted an anti-tumor immune response, whereas repeated chemotherapy cycles impaired anti-tumor immune functions. Though the amount of immune cells could recover after chemotherapy suspension, their anti-tumor functions were damaged by multiple rounds of chemotherapy. These findings also point towards early implementation of immunotherapy to improve the anti-tumor effect.

**Electronic supplementary material:**

The online version of this article (doi:10.1186/s12865-016-0167-7) contains supplementary material, which is available to authorized users.

## Background

Surgery, radiotherapy, chemotherapy and combined modality treatments designed to maximize anti-tumor effects with minimal toxicity to normal tissues have become standard clinical practice [1]. Clinically, chemotherapy schedules contain successive cycles for approximately half a year. However, drug resistance, metastasis and relapse of minimal residual disease (MRD) after therapies remain as significant challenges to cancer therapy [2].

In recent years, Kroemer and colleagues revealed the immunostimulatory functions of traditional chemotherapeutics. Reagents such as anthracyclines, cyclophosphamide and oxaliplatin can cause immunogenic cell death and trigger immune responses [3–5]. However, these chemotherapeutic reagents were studied using the model of a single administration [6, 7] or a limited number of administrations [8] rather than repeated cycles in the clinic. Clinical tumor samples are also collected and analyzed after chemotherapy, and the immune functions are reflected indirectly by the mRNA or protein levels of immune-related molecules [9].

Except for tumor inhibition, the toxicity of chemotherapy is often unavoidable. The obvious side effects of chemotherapies include nausea, vomiting, diarrhea, and increased infection rates, among others. The long-term toxicities are also recognized by increasing numbers of researchers. The stromal compartment of bone marrow can be remodeled after aplasia caused by chemotherapy [10, 11], but, hematopoietic reserve and function are usually chronically impaired [12, 13]. A study showed that administration of multiple cycles of cisplatin caused substantial sensory neuropathy and demonstrated that chemotherapy-induced nerve injury in the bone marrow of mice involves a crucial lesion that impairs hematopoietic regeneration [14]. Litterman et al. reported that high affinity responder lymphocytes that receive the strongest proliferative signal from vaccines experienced the greatest DNA damage response after alkylating chemotherapeutics, thus skewing the response toward lower affinity responders with inferior functional characteristics [15]. Clinically, adjuvant chemotherapy accelerates molecular aging of hematopoietic tissues [16]. Prigerson and colleagues found that chemotherapy use among patients with metastatic cancer whose cancers had progressed while receiving prior chemotherapy was not significantly related to longer survival [17]. They also showed that palliative chemotherapy did not improve quality of life near death (QOD) for patients with moderate or poor performance status and worsened QOD for patients with good performance status [18]. At the point of acquired drug resistance after chemotherapy, our lab proved that repeated 5-FU treatment could enrich slow-cycling tumor cells that are the source of tumor relapse and metastasis [19, 20]. Sun and colleagues collected prostate tumor samples before and after 4-cycle chemotherapy and showed that paracrine-acting secretory components such as WNT16B secreted by stromal cells after the initial round of chemotherapy in the prostate tumor microenvironment attenuated the effects of cytotoxic chemotherapy and promoted tumor drug resistance to subsequent cycles of cytotoxic therapy [21]. However, the immune status after different chemotherapy cycles has not been well studied.

Although chemotherapeutic drugs are administered for their selective toxicity to rapidly proliferating tumor cells, the adaptive immune response is also a highly proliferative process [15]. The immune status after each chemotherapy cycle is not absolutely clear, and the statuses are not compared. Chemotherapy could lead to the death of tumor cells and trigger an immune response against cancer cells. However, we speculate that if the tumor is not rejected completely, not only are the chemo-resistant cells enriched but also the specific anti-tumor immune cells can be impaired or destroyed after repeated cycles of chemotherapy.

In the current study, we developed a model of CT26 tumor-bearing mice treated with one to four cycles of 5-fluorouracil (5-FU). Tumor inhibition and overall survival (OS) after different cycles of treatment were observed. We attempted to explain the unimproved OS for more doses of chemotherapy from the point of view of the immune system. The amounts of different immune cells in the spleens and tumors were assayed after one and three cycles of 5-FU. The immune responses of spleen cells against CT26 were also analyzed, including proliferation, cytotoxicity, and released cytokines. We found that anti-tumor immune functions were impaired after three cycles of 5-FU (C3). Taking the immune system into account during chemotherapy and combining tumor-inhibitory and immunostimulatory chemotherapy with immunotherapy are rational approaches for future cancer treatments.

## Results

### Repeated cycles of 5-FU treatment inhibit tumor growth to a greater degree but do not improve OS compared with one cycle of 5-FU

**Fig. 1:**
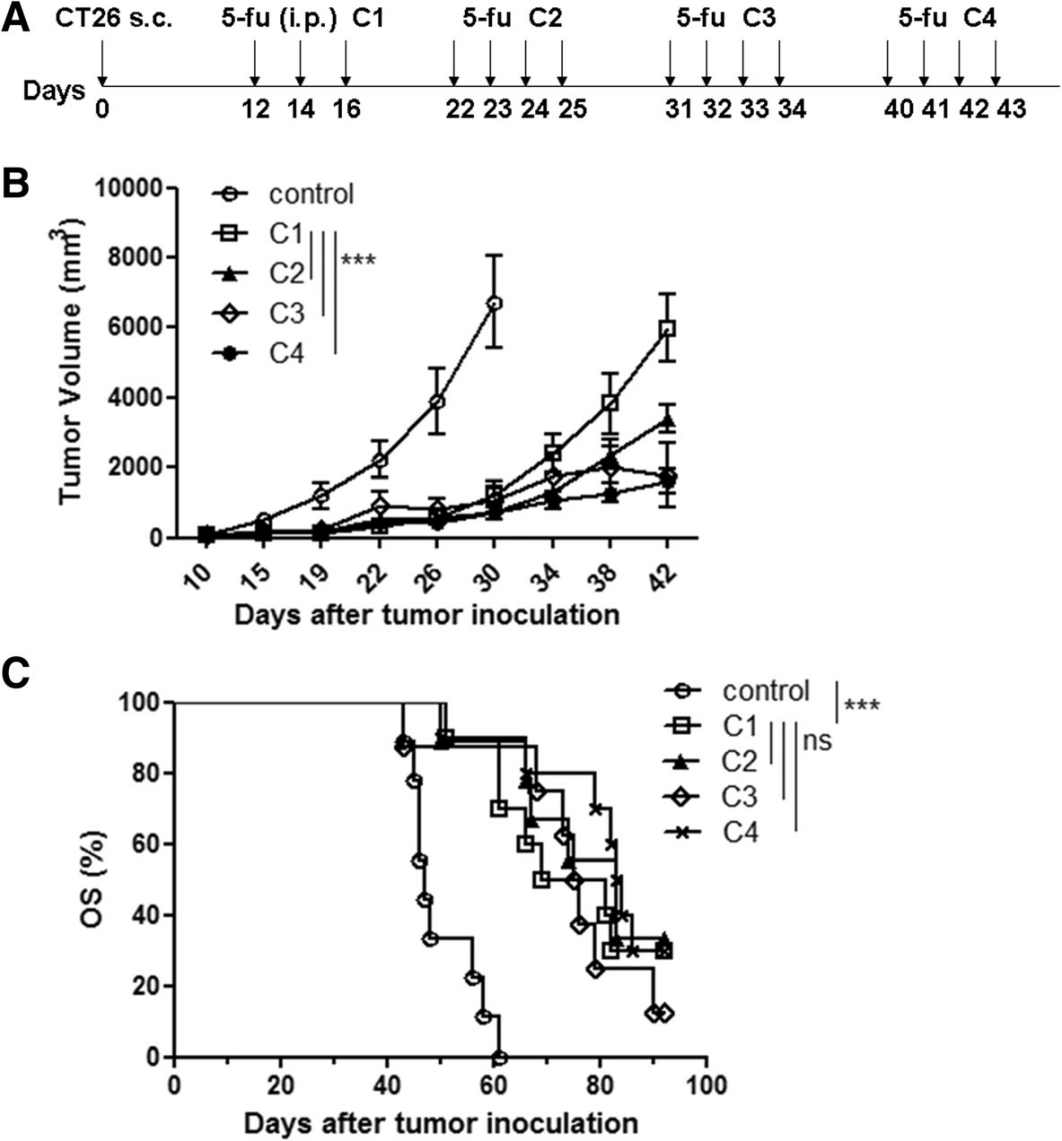
Increased cycles of 5-FU inhibit tumor growth to a greater degree but do not improve OS compared with one-cycle treatment. **a** 5-FU treatment schedule for CT26-bearing mice. A total of 3 × 10^5^ CT26 cells were injected s.c., and 5-FU (40 mg/kg) was administered at the indicated time point. **b** Tumor growth curve (data shown as the mean ± SEM) and **c** Kaplan-Meier survival analysis of mice treated with different cycles of 5-FU. Untreated tumor-bearing mice were used as a control (five to seven mice per group; results are representatives of at least two independent experiments). C1–C4, one to four cycles of 5-FU. Two-way ANOVA analysis for tumor volumes. Survival comparisons were calculated by the log-rank test. *, *p* < 0.05; **, *p* < 0.01; ***, *p* < 0.001; *ns*, no significance

Clinically, 5-FU based chemotherapy for treatment of colorectal cancer contains approximately eight cycles. To mimic the clinical schedule and investigate the therapeutic effect in our colon cancer model, CT26 cells were inoculated and tumor-bearing mice were treated with 5-FU for one to four cycles (Fig. 1a). Tumor volumes and OS were monitored. The maximum dosage for the first cycle is three injections of 5-FU (40 mg/kg). Four injections per cycle for the followed cycles were generally well tolerated. One cycle of 5-FU (C1) treatment could inhibit tumor growth compared with the untreated control group, and more than one cycle of treatment, namely two cycles (C2), three cycles (C3) and four cycles (C4), inhibited tumor growth to a greater degree than C1 (Fig. 1b; Additional file 1: Raw data Fig.1b). The OS of any of the treated groups was longer than the control group but not significantly different between treatment groups (Fig. 1c; Additional file 1: Raw data Fig 1c). Tumor growth was inhibited during chemotherapy but progressed after treatment suspension. Repeated cycles of 5-FU did not improve OS compared with one-cycle treatment. Non-durable immune responses against tumors after chemotherapy might be one of the reasons for unimproved OS, and therefore, we analyzed the immune status after different cycles of 5-FU in our model.

### 5-FU treatment does not decrease the amount of CD8 T-cells and NK cells

**Fig. 2:**
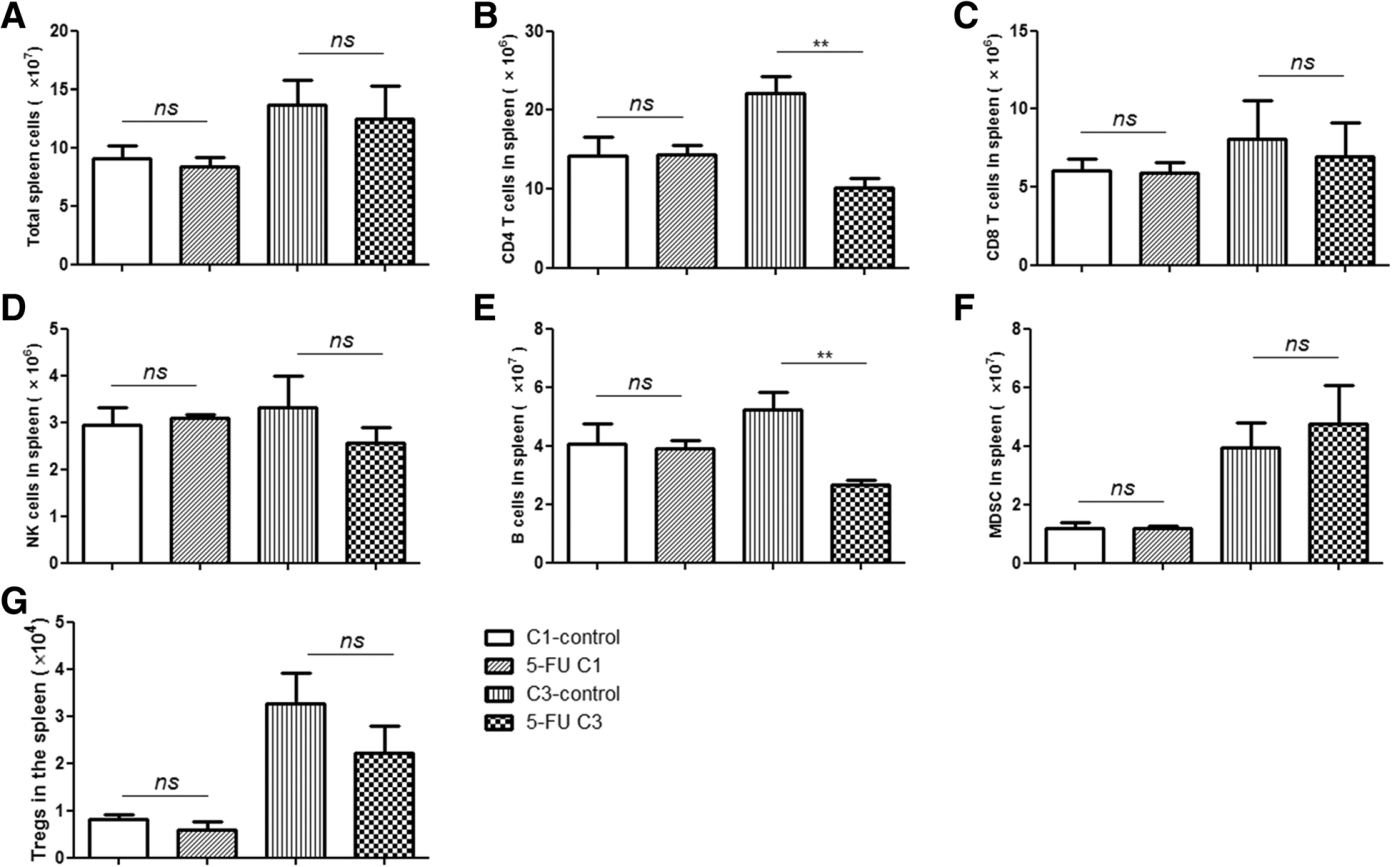
Amount of total spleen cells (**a**), CD4 T-cells (**b**), CD8 T-cells (**c**), NK cells (**d**), CD19^+^ B cells (**e**), MDSCs (**f**) and Treg cells (**g**) from 5- FU C1, C3 and their respective control groups were analyzed 7 days after the last 5-FU injection of each cycle. Student’s *t*-test was used to analyze the significance between groups. The results are representative of at least three independent experiments

**Fig. 3:**
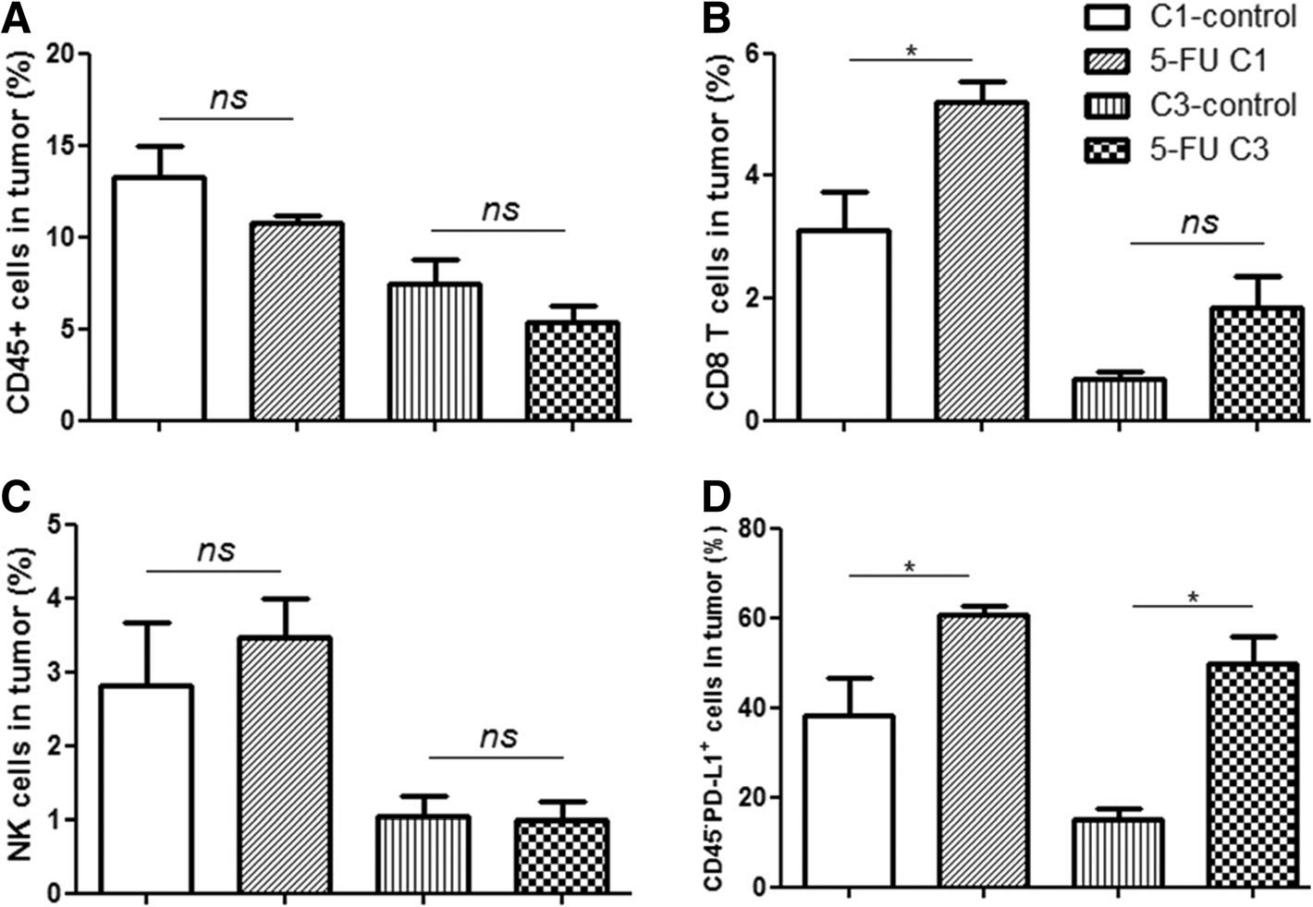
Percentage and PD-L1 expression of infiltrated immune cells in the tumor. Tumor tissues from 5-FU C1, C3 and control groups (each group, *n* = 3) were minced and digested. The percentages of CD45^+^ cells (**a**), CD8 T-cells (**b**) and NK cells (**c**) were analyzed by flow cytometry. PD-L1 expression in CD45^−^ cells (**d**) is shown. Student’s *t*-test was used to analyze the significance between groups

The blood cell count is an indicator for whether to continue chemotherapy in the clinic. We analyzed the absolute number of different immune cells in the spleens and their percentages in the tumors using flow cytometry. The spleen was usually enlarged with tumor growth (data not shown), but the total number of gradient-separated lymphocytes was not significantly different between the treated groups and their controls after a 7-day rest since last 5-FU injection (Fig. 2a; Additional file 2: Raw data Fig 2). CD4 T-cells (Fig. 2b) and B cells (Fig. 2e) were decreased after three cycles of 5-FU. The amount of CD8 T-cells (Fig. 2c) and NK cells (Fig. 2d) in the spleen did not decline significantly after treatment. Although 5-FU was reported to kill MDSCs, resulting in enhanced T cell-dependent antitumor immunity [22], the number of immunosuppressive cells, including MDSCs and Tregs, was not decreased significantly on day 7 after last 5-FU per cycle in our treatment groups (Fig. 2f, g). The percentages of immune subpopulations between the treatment and control groups were not significantly different except for B cells after C3 treatment (Additional file 3: Figure S1 and Additional file 4: Figure S2). Different immune subpopulations have different developmental pathways and recovery rates [23, 24]. B cells may not recover at the detected time point after three-cycle chemotherapy compared with other immune cells in our model. Alternatively, the recovery of B cells might have been impaired after 5-FU C3 treatment. The number of CD4 T-cells and B cells decreased, but cytokines primarily produced by CD4 T-cells (e.g., IL-10, IL-6 and TGF-β) or IgA secreted by B cells [25] were not decreased after C3 treatment (Additional file 5: Figure S4 and Additional file 6: Figure S5). In general, this observation might indicate that the acute myeloid suppression of 5-FU was probably relieved after the 7-day rest. In addition, immune cells that infiltrated into the tumor bed also should be considered. Because the infiltration of immune cells usually reached a peak 2 days after chemotherapy [26], we analyzed their distribution in the tumors on day 3 after last 5-FU injection. Compared with the control groups, the amount of CD45^+^ immune cells, including NK cells, was not changed significantly in the chemotherapy groups (Fig. 3a, c; Additional file 2: Raw data Fig 3). The percentage of tumor infiltrating CD8 T-cells was increased after 5-FU C1 treatment but not in the C3 group (Fig. 3b). Furthermore, the expression of PD-L1 (also called B7-H1) in tumor cells was analyzed, which could lead the anergy of activated T-cells [27]. In our treatment model, a greater number of non-immune cells (CD45^−^ cells, primarily including tumor cells and fibroblast cells) expressed PD-L1 after chemotherapy (Fig. 3d). This observation might imply that anti-tumor immune functions were repressed, and the tumor could relapse after suspension of 5-FU treatment. Elevated PD-L1 after 5-FU C1 treatment also indicated that early intervention of unanticipated aspects of chemotherapy by immune strategies might be needed.

### In vitro proliferation and cytotoxicity against CT26 are impaired after 5-FU C3 treatment

**Fig. 4:**
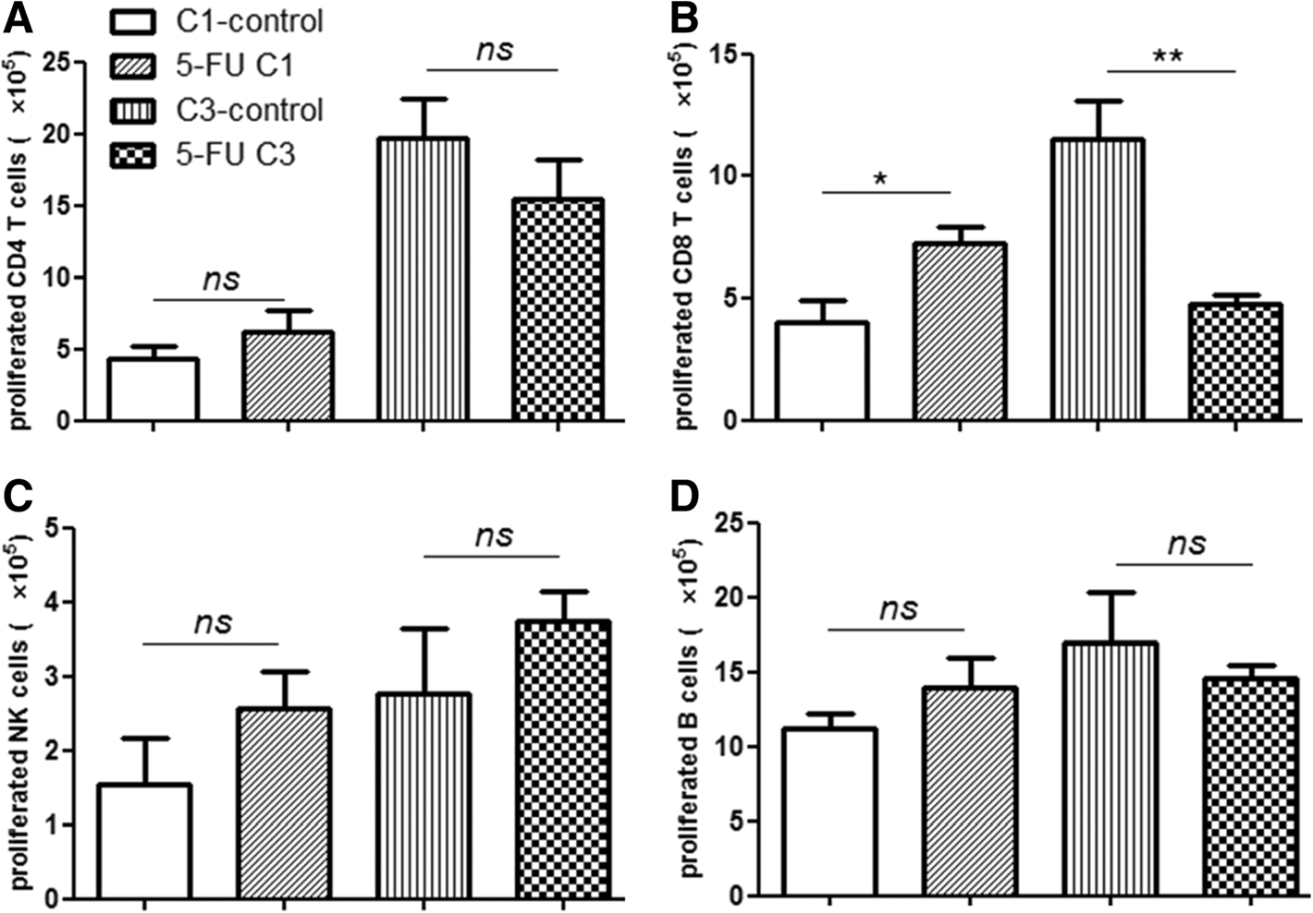
Absolute number of proliferated immune cells after 5-FU C1 and C3. Proliferated immune cells were detected by CFSE assay, and the absolute numbers of proliferated CD4 T-cells (**a**), CD8 T-cells (**b**), NK cells (**c**) and B cells (**d**) were calculated. The significance between groups was analyzed by Student’s *t*-test. The results are representatives of at least three independent experiments

To identify specific anti-tumor immune functions after C1 and C3, the proliferation and cytotoxicity of spleen cells against CT26 were assayed. First, we investigated the proliferation of lymphocytes from different treatment groups using CFSE assays and analyzed the absolute number of proliferated lymphocytes against CT26 (the definition of proliferated cells is illustrated in Additional file 7: Figure S3). The total proliferated CD8 T-cells increased after C1 treatment but decreased after C3 compared with untreated groups, respectively (Fig. 4b; Additional file 2: Raw data Fig 4). The proliferated CD8 T-cells were tumor-specific clones because the proliferation was stimulated by CT26. Other cells, including CD4 T-cells (Fig. 4a), NK cells (Fig. 4c) and B cells (Fig. 4d), were not significantly different between the treated and control groups.

**Fig. 5:**
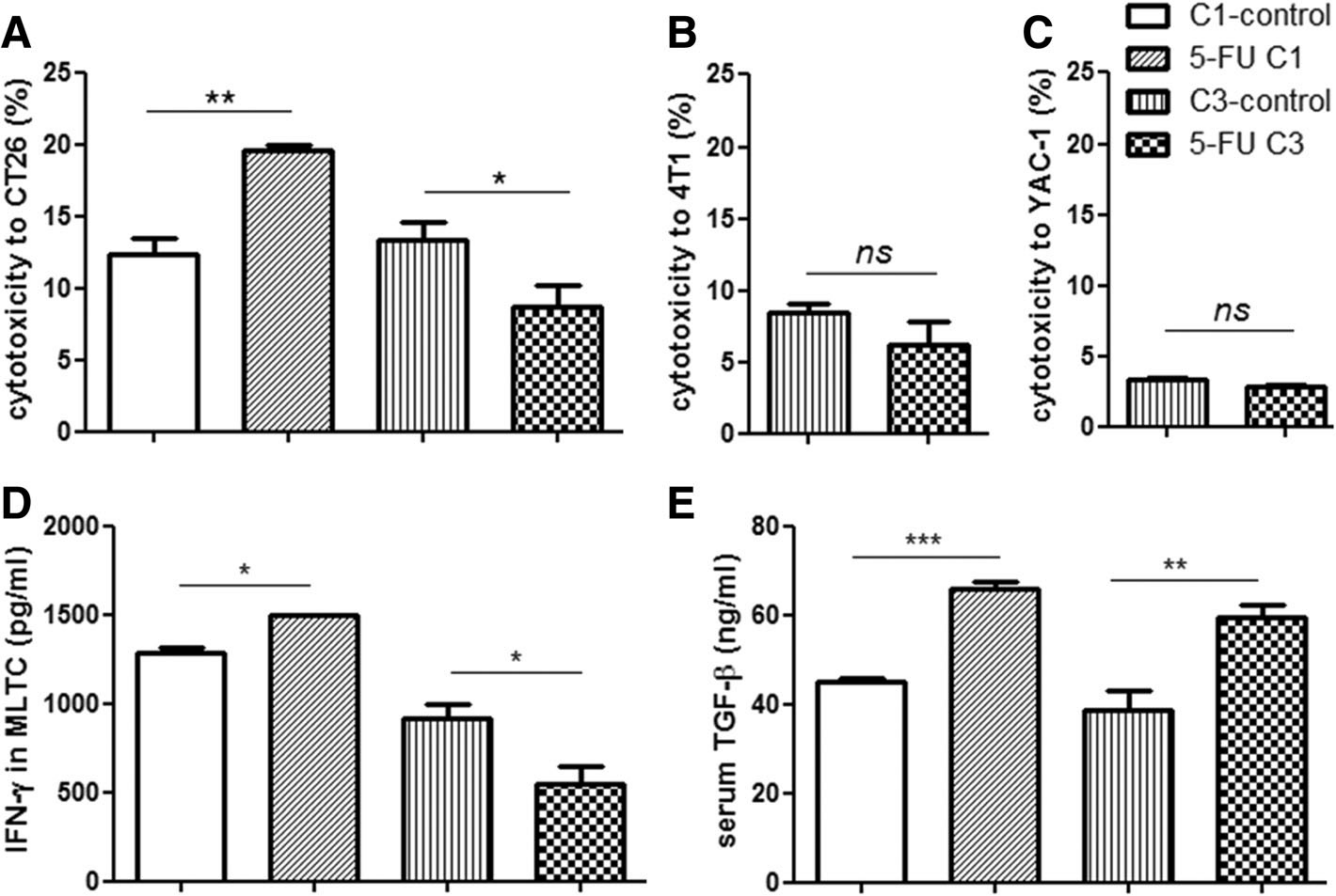
Cytotoxicity and cytokine production after 5-FU C1 and C3 treatment. **a** Cytotoxicity against CT26 using CFSE-PI staining-based flow cytometry. Spleen cells from treatment and control groups as effectors were incubated with CFSE-stained CT26 cells (Fig. 5a) at an effector:target (E:T) ratio of 25:1. CFSE and PI positive cells represent killed target cells, and the cytotoxicity was calculated. **b** Cytotoxicity against 4T1 cells, and **c** cytotoxicity against YAC-1 cells at the E:T ratio of 25:1. **d** IFN-γ production by spleen cells in the MLTC assay. The supernatant of MLTC was collected on day 3, and the IFN-γ concentration was analyzed by ELISA. **e** Serum TGF-β was quantified by ELISA. Serum was collected from C1, C3 and control groups (*n* = 3) on day 7 after the last 5-FU injection. Student’s *t*-test was used to analyze the significance between groups. The experiments were replicated at least twice with similar results

Second, we determined the cytotoxicity of spleen cells against CT26. Lymphocytes from the 5-FU C1 and control group showed 19.5(±0.5) % and 12.4(±1.1) % cytotoxicity against CT26, respectively. In contrast, lymphocytes from 5-FU C3 showed only 8.7(±1.5) % cytotoxicity against CT26 cells, whereas the control group remained at 13.3(±1.3) % (Fig. 5a; A dditional file 8: Raw data Fig 5). The cytotoxicity of lymphocytes from C3 was the lowest. The changes of cytotoxicity was CT26 cell-specific because no significant difference of cytotoxicity was observed between the C3 treated and control groups against 4 T1 cells (Fig. 5b), which is from the synergetic Balb/C mice. This cytotoxicity was executed primarily by CD8 T-cells because the cytotoxicity against YAC-1 (NK sensitive target cells) was approximately 3 % in both groups (Fig. 5c). The decreased cytotoxicity was due to impaired anti-tumor responses after 5-FU C3 treatment because the ratios of CD8 T-cells were not significantly different between the treated and control groups (Additional file 4: Figure S2B).

Third, the culture supernatants of MLTC from different groups were collected to examine the released cytokines using ELISA assays. IFN-γis a well-known anti-tumor cytokine, and its content was higher in 5-FU C1 but decreased in C3 compared with their control groups. Tumor-promoted cytokines such as IL-10, IL-6, TGF-βand IgA in the medium of MLTC were not reduced after 5-FU C3 treatment (Additional file 5: Figure S4A-D; Additional file 8: Raw data Fig S4). Cytokines in serum were also determined by ELISA assays. TGF-β, an immunosuppressive and tumor-promoted cytokine, was increased after 5-FU treatment (Fig. 5e), similar to radiation- and doxorubicin-treated tumor-bearing MMTV/PyVmT mice [28]. The concentration of IL-6 was improved whereas IL-10 and IgA were not changed after 5-FU C3 (Additional file 6: Figure S5A–C; Additional file 8: Raw data Fig S5). Increased IL-6 after 5-FU C3 might help to promote chronic inflammation and residual tumor survival, which was a negative factor in anti-tumor responses. IFN-γ in the serum was too low to be detected (data not shown).

### One-cycle 5-FU combined with CIKs and PD-L1 antibodies improves therapeutic efficacy in vivo

**Fig. 6:**
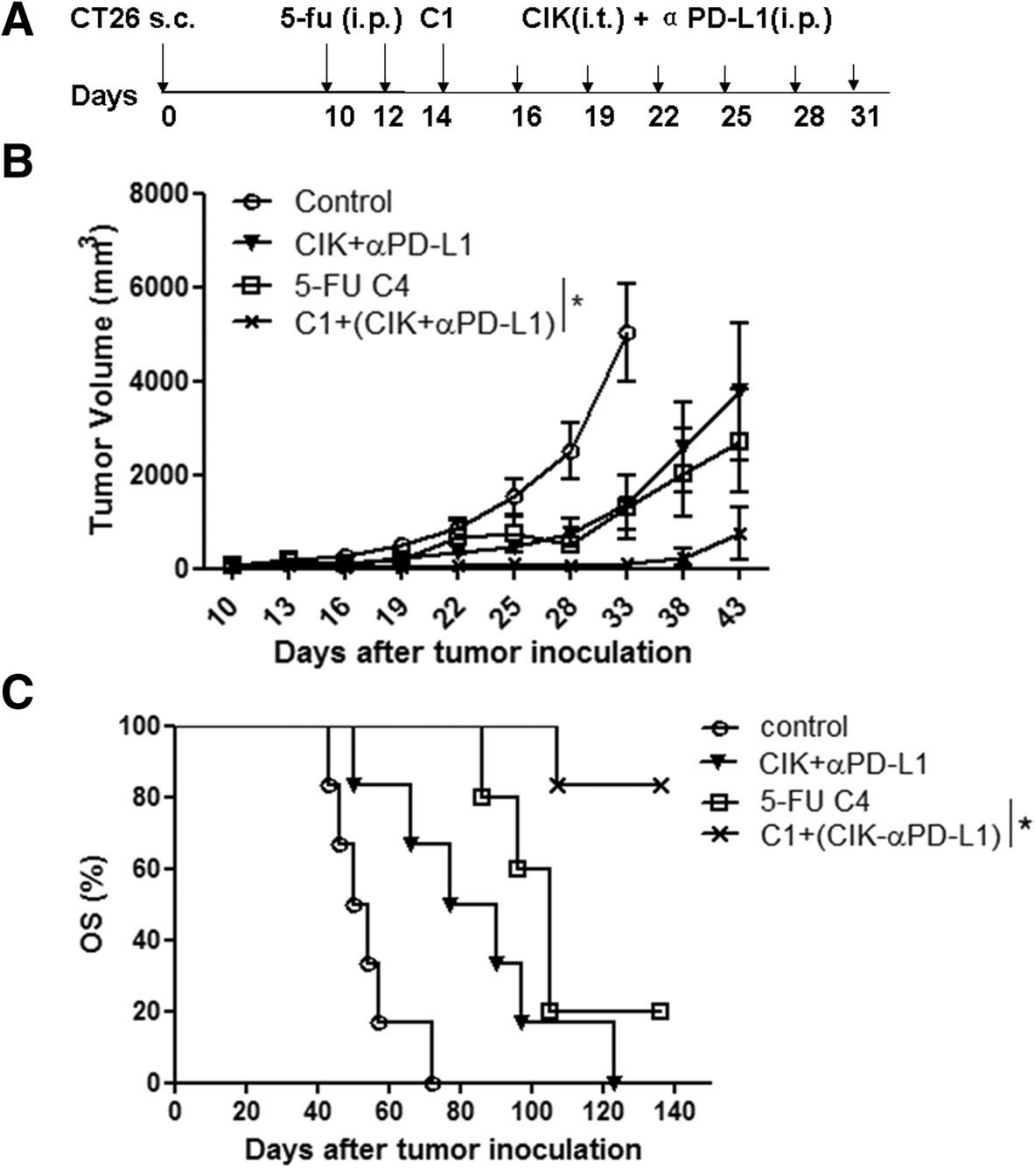
One-cycle 5-FU combined with CIKs and PD-L1 antibodies improves therapeutic effect in vivo. **a** The chemo-immunotherapy schedule of CT26 tumor-bearing mice. Second day after 5-FU C1 last injection, 1 × 10^7^ CIKs were injected into tumor and 200 μg αPD-L1 antibodies were injected intraperitoneally per mouse every 3 days for six times. **b** Tumor growth curve (data shown as the mean ± SEM) and **c** Kaplan-Meier survival analysis of control, 5-FU C4, immunotherapy (CIKs andαPD-L1) and chemo-immunotherapy. Five to six mice per group and the experiments replicated at least twice with similar results. Two-way ANOVA analysis for tumor volumes and log-rank test for survival comparisons

A single round of 5-FU promoted the proliferation and cytotoxicity of CD8T-cells, whereas repeated cycles of chemotherapy impaired anti-tumor immune functions. The impaired tumor-specific responses might be a critical reason underlying non-durable anti-tumor activity. Therefore, the immediate combination of immunotherapy rather than repeated chemotherapy may help to improve and prolong the anti-tumor effect in cancer treatment. Because PD-L1 was more highly expressed after chemotherapy (Fig. 3d), PD-L1 monoclonal antibodies (αPD-L1) and killer cells (CIKs) were administered after 5-FU C1 (Fig. 6a). As shown in Fig. 6b (Additional file 9 : Raw data Fig 6 ), the combined therapy displayed a greater inhibition of tumor growth compared with chemotherapy or immunotherapy alone. The OS of the combination treatment group was also improved (Fig. 6c). Indeed, half of the mice in the combined group were tumor-free for more than 6 weeks after tumor regression (data not shown).

## Discussion

5-FU is an analog of uracil that operates as an anti-metabolite by inhibiting thymidylate synthase [29] and is a widely used chemotherapeutic agent for colorectal cancer. However adverse effects including life-threatening mucositis or bone marrow suppression occur in approximately 11 % of patients on infusion therapy and 25 % with bolus therapy [30]. In our CT26 tumor-bearing murine model, we imitated the clinical chemotherapy schedule for the cycles and examined the immune functions after one- and three-cycle treatment rather than only final evaluation of immune status after chemotherapy [6, 8, 22].

Prolonged OS was observed for 5-FU treated tumor-bearing mice. Although two or more cycles of treatment inhibited tumor growth to a greater degree, they did not improve OS compared with C1. After C1 treatment, more CD8 T-cells infiltrated tumors. Improved in vitro activity, including enhanced cytotoxicity, more proliferated CD8 T-cells and IFN-γsecretion of spleen cells were observed after C1 treatment. Then after C2 treatment, no improvements of cytotoxicity and proliferation of spleen cells against CT26 were observed (Additional file 10: Figure S6; Additional file 8: Raw data S6 a; Additional file 2: Raw data S6b-c). Moreover, the proliferation and cytotoxicity were impaired after C3 treatment in contrast to control. Increased immune suppressive factors such as TGF-β, IL-10 and PD-L1 after C1 treatment implied that implementation of immunotherapy against these factors should occur as early as possible to improve the anti-tumor effects of chemotherapy in certain types of tumors. And impaired anti-tumor immune functions after C3 might indicate that immediate combination of immunotherapies rather than repeated chemotherapies may be a preferred choice for persistent anti-tumor treatment to improve anti-tumor effect. Our study showed that combining CIKs and anti-PD-L1 with one-cycle 5-FU performed better than four-cycle 5-FU in cancer treatment. The cured mice after chemo-immunotherapy were rechallenged with CT26 cells. None of these mice had tumor formation (Additional file 11: Figure S7; Additional file 9: Raw data S7), which indicated that 5-FU C1 combined with CIKs and anti-PD-L1 induced a specific memory immune response in vivo.

The immune-regulatory roles of 5-FU were previously reported by Vincent and colleagues, who demonstrated that treatment of EL4 tumor-bearing mice with 5-FU led to decreased MDSC in the spleens and increased IFN-γproduction by tumor-specific CD8^+^ T-cells that infiltrated the tumor [22]. In their study, only one 5-FU treatment at 50 mg/kg was performed, and the results were analyzed 5 days later. The work from the same lab stated that 5-FU also induces activation of NLRP3 in dying MDSC, leading to secretion of IL-1β, elicitation of TH17 cells, IL-17 production and tumor growth following increased angiogenesis [31]. Other studies proved that the tumor-specific immune response was enhanced by 5-FU when 5-FU was combined with a tumor vaccine [32, 33]. We confirmed improvement in the anti-tumor immune response after 5-FU C1 treatment. Additionally, we revealed that the immune-related benefits of 5-FU treatment were lost after repeated 5-FU cycles.

The success of cancer treatment cannot be achieved without activated anti-tumor immune functions [34, 35]. Immune status should be taken into account for unimproved OS after cycles of chemotherapy. Mackall explained that myeloablative therapy, dose-intensive alkylating agents, purine nucleoside analogs and corticosteroids substantially increase the risk of therapy-induced immuno-suppression [36]. Mackall and colleagues noted that the total CD8 T-cell number recovered relatively quickly in both children and adults post-therapy; however, functional CD8^+^ subset (e.g., CD8^+^ CD28^+^) disruptions often remained for a prolonged period together with the difficulty of TCR repertoire diversity restoration. NK cells appeared to be relatively resistant to cytotoxic antineoplastic therapy [23, 24]. Another study reported that the functional T-cell responses were normal because the proliferation of T-cells against common antigens (like antigen from CMV virus) was similar to those of the healthy controls [37]. However, whether the proliferation against common antigens can reflect functional T-cells against tumor antigens is not quite clear. According to Litterman’s study, highly proliferative lymphocytes experienced the greatest DNA damage after alkylating chemotherapeutics [15]. We hypothesize that activated and proliferating anti-tumor immune cells are damaged over and over again by successive cycles of chemotherapy. Although immune homeostasis can be reconstructed after chemotherapy, tumor-specific clones are more difficult to restore and may remain depleted for a prolonged period, especially in adults [36]. Our CT26 cell grafted model might not accurately reflect tumor genesis, but it is acceptable as a tumor model for monitoring of tumor inhibition by chemotherapy and evaluation of disease progression. Whole tumor cell antigens are available, and specific anti-tumor immune responses could be detected. The impaired proliferation and cytotoxicity of CD8 T-cells and unaffected NK cells in our 5-FU C3 treatment group were consistent with Mackall’s conclusions.

Clinically, 5-FU is commonly used at lower dosage and combined with other agents, such as leucovorin and oxaliplatin, to improve anti-tumor effects and minimize the toxicity [38, 39]. In our experiments, only 5-FU was used to treat colon cancer in vivo. It might be possible to combine two or more agents to improve the chemotherapy effects and analyze their immune responses. In addition, whether immune functions are impaired similarly in other cancers treated with different chemotherapeutic agents is a question that requires further investigation, and this work will more useful if it is confirmed by clinical samples.

## Conclusions

Our multi-cycle chemotherapy model suggested that repeated cycles of chemotherapy harmed the specific anti-tumor immune responses in contrast to the chemo-immunotherapeutic role of one-cycle 5-FU treatment. Immediate combination of anti-PD-L1 and CIKs increased therapeutic efficacy. In the future, conditional chemotherapy combined with early introduction of immunotherapy, such as immune checkpoint blockades, vaccines and adoptive transfer of T-cells instead of repeated chemotherapy, is a promising approach to restoring anti-tumor immune system and improving the efficacy of cancer treatment.

## Methods

### Mice and cell lines

Male 7-week-old Balb/C mice purchased from Vital River Laboratory Animal Technology Co. Ltd. (Beijing, China) were maintained under specific pathogen-free conditions in the animal facility of Cancer Institute, Chinese Academy of Medical Sciences (CAMS). All procedures for animal experiments were approved by the Institutional Animal Use and Care Committee of CAMS. CT26 cells (a colon cancer cell line from Balb/C mice) were obtained from the Cell Resource Center, Peking Union Medical College (which is the headquarters of National Infrastructure of Cell Line Resource, NSTI) on Jan 10, 2015. The cell line was determined to be free of mycoplasma contamination by PCR and culture. Its species origin was confirmed with PCR. The identity of the cell line was authenticated with STR profiling (FBI, CODIS). All results can be viewed on the website (http://www.cellresource.cn/). 4 T1 cells (a breast cancer cell line from Balb/C mice) and YAC-1 (a mouse lymphoma cell line) cells were purchased from American Type Culture Collection (ATCC), (Manassas, VA, USA) and maintained in our laboratory. CT26 and YAC-1 cells were cultured in RPMI 1640 medium, and 4 T1 cells were cultured in DMEM/F12 medium (Hyclone, Thermo Fisher Scientific, USA). Cells were maintained in basic medium supplemented with 10 % FBS and penicillin/streptomycin at 37 °C in a humidified atmosphere of 5 % CO_2_.

### Tumor models and treatments

CT26 cells (3 × 10^5^) were inoculated subcutaneously (s.c.) in the right flanks of Balb/C mice. Tumor growth was monitored every 3–4 days by palpation. Tumor diameters were measured twice weekly and used to calculate tumor volumes, as described previously [19]. Mouse survival was monitored every other day.

Approximately 2 weeks after tumor inoculation, 5-FU (40 mg/kg) was injected intraperitoneally (i.p.) every other day for a total of three injections for the first cycle treatment (C1). A week after the last 5-FU injection, 5-FU 40 mg/kg was injected every day for a total of four injections for the second cycle (C2) treatment. The third cycle (C3) and fourth cycle (C4) were as same as C2.

For immunotherapy, 1 × 10^7^ CIK cells (200 μl) and anti-PD-L1 mAb (200 μg/mouse) were injected into tumor and peritoneally respectively after C1 at a 3-day interval for six injections.

### Cell isolation and flow cytometry analysis

Splenic cells were isolated by gradient centrifugation using lymphocyte separation medium (Dakewe Biotech, Shenzhen, China). Tumor tissues were minced and digested in RPMI containing 2 % FBS, 1 mg/ml type IV collagenase (Sigma Aldrich) and 300 U/ml DNase I (Sigma Aldrich) for 2 h at 37 °C, and passed through a cell strainer to achieve cell suspension.

For surface staining, cells were suspended in staining buffer and incubated for 30 min at 4 °C in the dark with fluorophore-conjugated anti-mouse mAbs: APC-anti-CD3, PerCP-Cy5.5-anti-CD4, PE-anti-CD8, PE-anti-CD19, PE-anti-CD49b, PE-Cy5-anti-CD11b, PE-anti-Gr-1, and APC-anti-PD-L1. For intracellular Foxp3 staining, cells were fixed and permeabilized according to the manufacturer’s protocol and incubated with PE-conjugated anti-mouse Foxp3 antibodies for 30 min at 4 °C in the dark. All antibodies and Foxp3 fixation/permeabilization kit were purchased from Biolegend. Cells were acquired by a flow cytometer (BD LSRII) and analyzed using Flowjo software.

### CFSE proliferation assay

To analyze the proliferation of different subsets of lymphocytes, separated splenic cells were labeled with CFSE (Life Technologies) according to the manufacturer’s protocol and incubated with mytomycin C (MMC)-treated CT26 cells at a responder:stimulator (R:S) ratio of 10:1. Three days later, cells were collected and stained with the mixture of fluorophore-conjugated anti-mouse mAbs as indicated and analyzed by flow cytometry.

### In vitro cytotoxic assays

Seven days after the last 5-FU injection of C1 and C3, splenic cells from treated and non-treated tumor-bearing mice were prepared as effector cells. CT26, 4 T1 and YAC-1 were used as the targeted cells, respectively. As described previously [19], target cells were labeled with CFSE and mixed with effector cells at an effector:target (E:T) ratio of 25:1. The mixed cells were spun down and incubated at 37 °C for 4 h. PI (Sigma Aldrich) was added for DNA labeling of dead cells at the end of the incubation period. Samples were analyzed by flow cytometer within 30 min.

### Determination of the concentrations of IFN-γ and TGF-β by ELISA

As described above [19], splenic cells from differently treated and non-treated tumor-bearing mice were incubated with MMC inactivated CT26 (alive but not proliferative) at a R:S ratio of 10:1. The supernatant of the mixed lymphocyte and tumor cell culture (MLTC) was collected on day 3, and the concentrations of IFN-γ were determined using the mouse IFN-γ ELISA kit (Dakewe Biotech, Shenzhen, China). Serum from treated and control mice were collected and detected via the TGF-β ELISA kit (NeoBioscience Ltd., Beijing, China).

### Generation of cytokine induced killer (CIK) cells and anti-mouse PD-L1 monoclonal antibodies

CIKs were generated as previously described [40]. Briefly, lymphocytes from CT26-bearing mice were stimulated with recombinant mouse IFN-γ (1000 U/ml; Peprotech) for 24 h, then transferred to anti-CD3 (145-2C11; Biolegend) pre-coated tissue-culture flasks, and stimulated with 300 IU/ml recombinant human IL-2 every 3 days until cells were harvested on day 10. Anti-PD-L1 (clone 10B5) hybridoma was kindly provided by Shengdian Wang Lab (Institute of Biophysics, Chinese Academy of Sciences, Beijing, China). Anti-mouse PD-L1 monoclonal antibodies (αPD-L1) were purified from ascites of nude mice [41].

### Statistical analysis

The statistical significance between groups was determined by Student’s *t*-test, and tumor volumes were analyzed using two-way ANOVA. The Kaplan-Meier survival plot was assessed for significance using the log-rank test. All statistical analyses were performed using GraphPad Prism software version 5 (GraphPad Software, Inc.), and *P* < 0.05 was considered significant.

## Additional files

Additional file 1: Raw data Figure 1B and 1C Tumor volumes and mice survivals of differently treated groups, namely control, C1, C2, C3, and C4 groups. (XLSX 19 kb) Additional file 2: Raw data Figure 2, 3, 4, S2, and S6B-C The numbers (Figure 2) and percentages (Figure S2) of different immune cells in spleens, the percentages of different immune cells in tumors (Figure 3), and the numbers of proliferated immune cell in spleens (Figure 4, S6B and S6C), of differently treated groups. (XLSX 15 kb) Additional file 3: Figure S1. The flow cytometry dot plots of different immune cells from the 5-FU C1, C3 and control groups. Spleen cells from different groups were separated 7 days after the last 5-FU injection of each cycle. Immune cells were stained with fluorophore-conjugated antibodies and analyzed by a FACS Calibur flow cytometer. CD3-positive and CD4-positive cells are CD4 T-cells. CD3-positive and CD8-positive cells are CD8 T-cells. CD3-negative and DX5-positive cells are NK cells. Gr-1-positive and CD11b-positive cells are MDSCs. CD3-negative and CD19-positive cells are B cells. CD4-positive and CD25-positive cells are Tregs. (TIF 5348 kb) Additional file 4: Figure S2. Statistical data of percentages of CD4 T-cells (A), CD8 T-cells (B), NK cells (C), CD19^+^ B cells (D), MDSCs (E) and Treg cells (F) from the 5-FU C1, C3 and control groups analyzed 7 days after the last 5-FU injection of each cycle. Student’s *t*-test was used to analyze the significance between groups. The results are representative of at least three independent experiments. (TIF 1946 kb) Additional file 5: Figure S4. (A) IL-10, (B) IL-6, (C) TGF-β, and (D) IgA secreted by spleen cells in the MLTC assay. The supernatant of MLTC was collected on day 3 and analyzed by ELISA. Student’s *t*-test was used to analyze the significance between groups. The experiments were replicated at least twice with similar results. (TIF 1023 kb) Additional file 6: Figure S5. (A) IL-10, (B) IL-6, and (C) IgA concentrations in serum of 5-FU treated and control mice. Serum was collected on day 7 after the last 5-FU injection, and cytokines were quantified by ELISA. Student’s *t*-test was used to analyze the significance between groups. The experiments were replicated at least twice with similar results. (TIF 943 kb) Additional file 7: Figure S3. Determination of proliferated CD8 T-cells by CFSE assay. CD8 T-cells were gated and calculated using their absolute number multiplied by their proliferated percentage (the cells with reduced CFSE expression were proliferated cells) to calculate the proliferated cell numbers. Proliferated CD4 T-cells, NK cells and B cells were detected in the same manner. (TIF 635 kb) Additional file 8: Raw data Figure 5, S4, S5, and S6 Cytotoxicity of spleen cells against CT26, 4 T1 and YAC-1 (Figure 5A-C, and S6A); and concentrations of different cytokines in the supernatants of MTLC (Figure 5D and S4) and serum (Figure 5E and S5). (XLSX 12 kb) Additional file 9: Raw data Figure 6B, 6C, and S7 Tumor volumes (Figure 6B), mice survival (Figure 6C) of differently treated groups, namely control, CIK+ α PD-L1, 5-FU C4, and C1+ (CIK+ α PD-L1); and rechallenge data (Figure S7) of tumor-free mice after chemo-immunotherapy. (XLSX 17 kb) Additional file 10: Figure S6. In vitro immune functions of spleen cells against CT26 after 5-FU C2 treatment. (A) Cytotoxicity of spleen cells against CT26 from C2 and control groups were analyzed at the E:T ratio of 25:1. Proliferation of CD8 T cells (B) and NK cells (C) against CT26 were determined at the R:S ratio of 10:1 by CFSE assay. (TIF 618 kb) Additional file 11: Figure S7. Cured mice after chemo-immunotheapy (i.e., C1+ (CIK + α PD-L1)) were resistant to CT26 rechallenge. One month after final administration of CIKs and PD-L1 antibodies, cured mice and naïve control mice were inoculated with 1 × 10^6^ CT26 on the opposite side. Tumor formation were monitored and calculated. (TIF 541 kb)

## Abbreviations

5-FU: 5-fluorouracil
CFSE: Carboxy fluorescein diacetate succinimidyl ester
CIK: Cytokine induced killer
IFN-γ: Interferon-gamma
IL-6: Interleukin-6
IL-10: Interleukin-10
MDSC: Myeloid-derived suppressor cell
PD-L1: Programmed death-ligand 1
PI: Propidium Iodide
TGF-β: Transforming growth factor
