# Supplementary figures and images for: Repeated cycles of 5-fluorouracil chemotherapy impaired anti-tumor functions of cytotoxic T cells in a CT26 tumor-bearing mouse model
Source: BMC Immunol. 2016 Sep 20;17:29. doi: 10.1186/s12865-016-0167-7 (PMC5028929; doi:10.1186/s12865-016-0167-7)

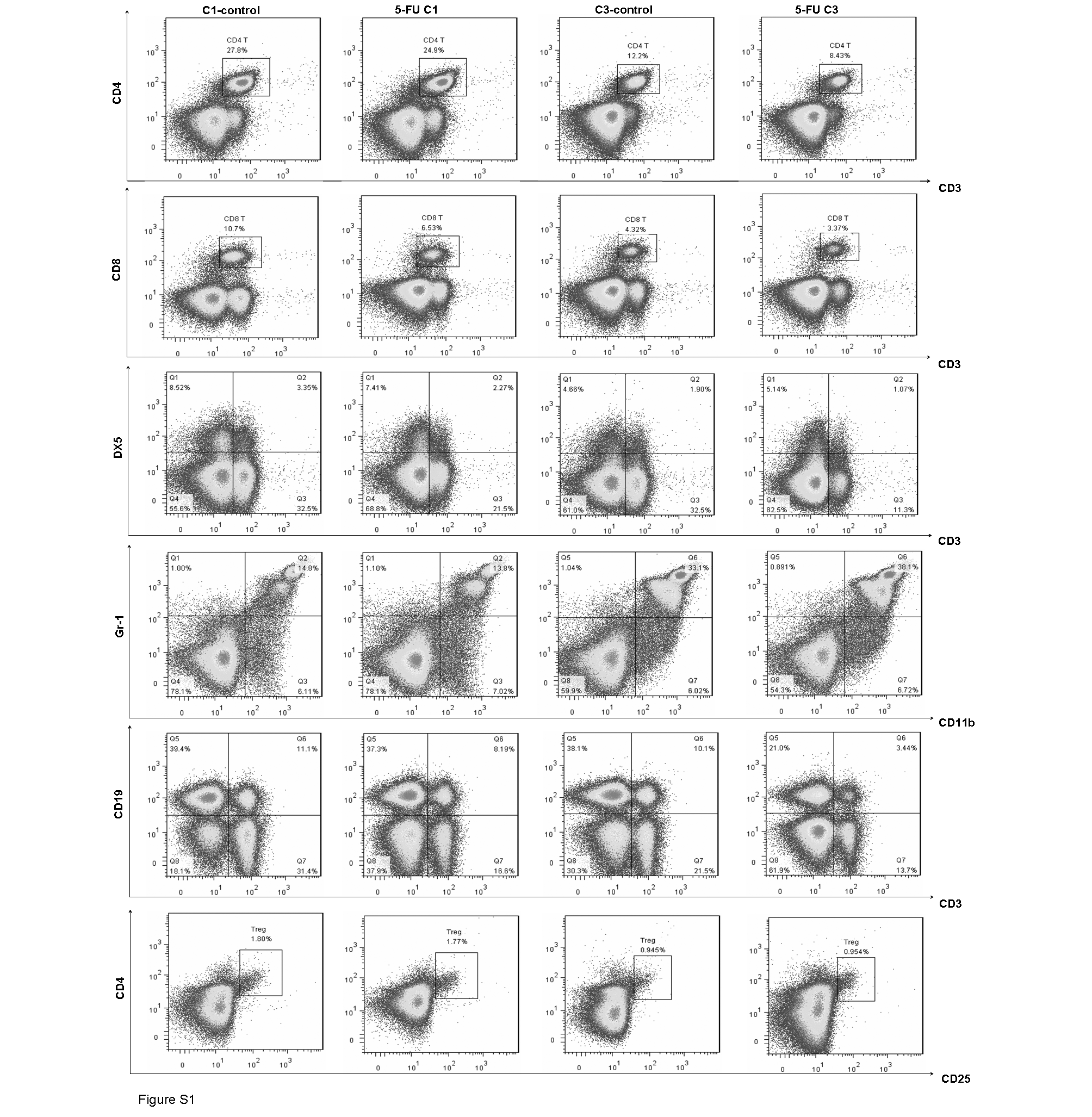

Supplement: Additional file 3: Figure S1. — The flow cytometry dot plots of different immune cells from the 5-FU C1, C3 and control groups. Spleen cells from different groups were separated 7 days after the last 5-FU injection of each cycle. Immune cells were stained with fluorophore-conjugated antibodies and analyzed by a FACS Calibur flow cytometer. CD3-positive and CD4-positive cells are CD4 T-cells. CD3-positive and CD8-positive cells are CD8 T-cells. CD3-negative and DX5-positive cells are NK cells. Gr-1-positive and CD11b-positive cells are MDSCs. CD3-negative and CD19-positive cells are B cells. CD4-positive and CD25-positive cells are Tregs. (TIF 5348 kb) [file 12865_2016_167_MOESM3_ESM.tif]

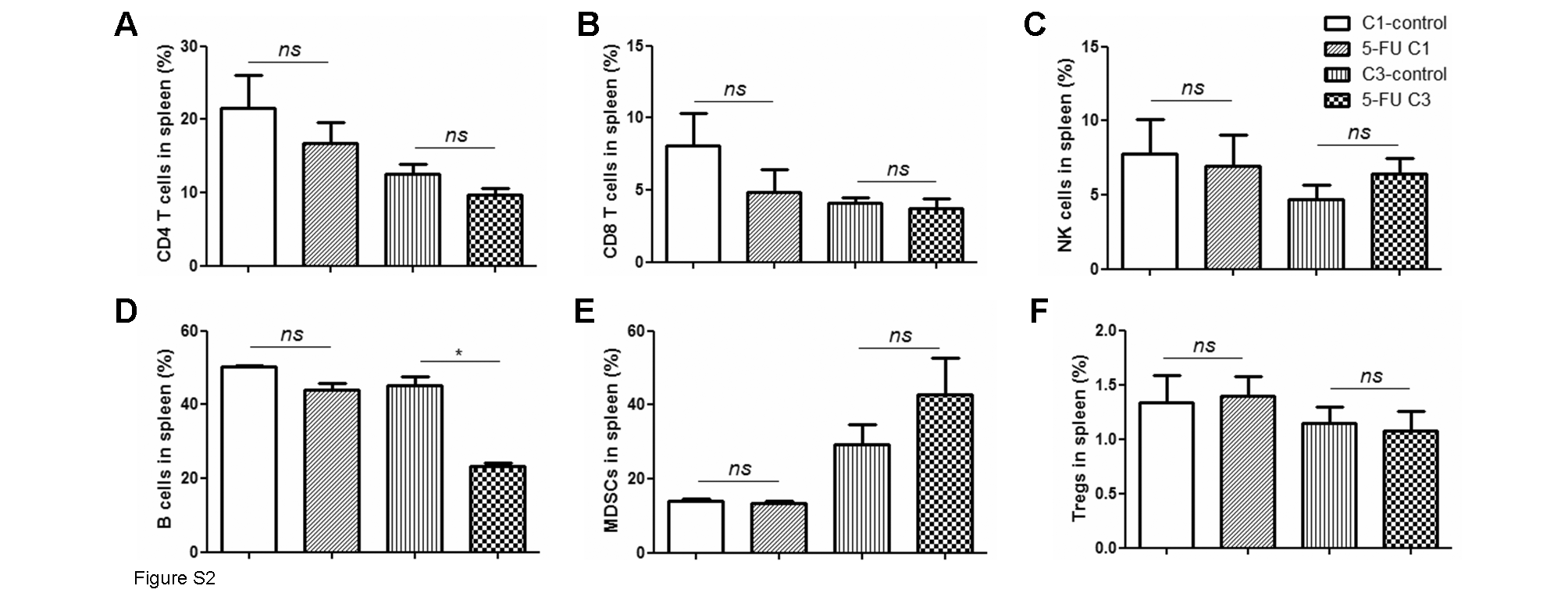

Supplement: Additional file 4: Figure S2. — Statistical data of percentages of CD4 T-cells (A), CD8 T-cells (B), NK cells (C), CD19+ B cells (D), MDSCs (E) and Treg cells (F) from the 5-FU C1, C3 and control groups analyzed 7 days after the last 5-FU injection of each cycle. Student’s t-test was used to analyze the significance between groups. The results are representative of at least three independent experiments. (TIF 1946 kb) [file 12865_2016_167_MOESM4_ESM.tif]

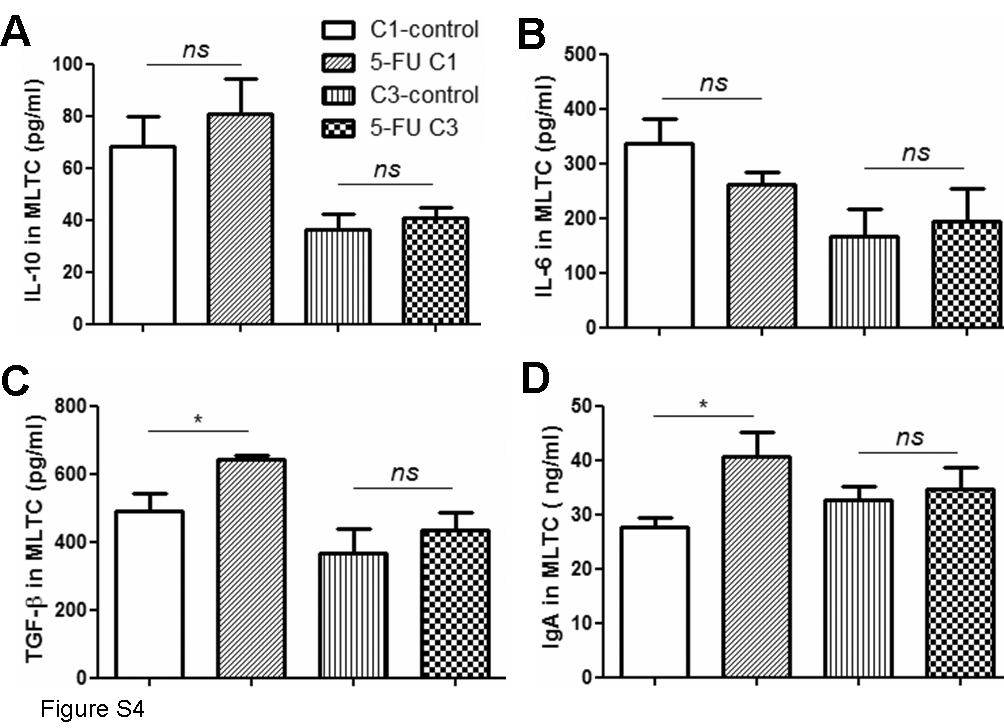

Supplement: Additional file 5: Figure S4. — (A) IL-10, (B) IL-6, (C) TGF-β, and (D) IgA secreted by spleen cells in the MLTC assay. The supernatant of MLTC was collected on day 3 and analyzed by ELISA. Student’s t-test was used to analyze the significance between groups. The experiments were replicated at least twice with similar results. (TIF 1023 kb) [file 12865_2016_167_MOESM5_ESM.tif]

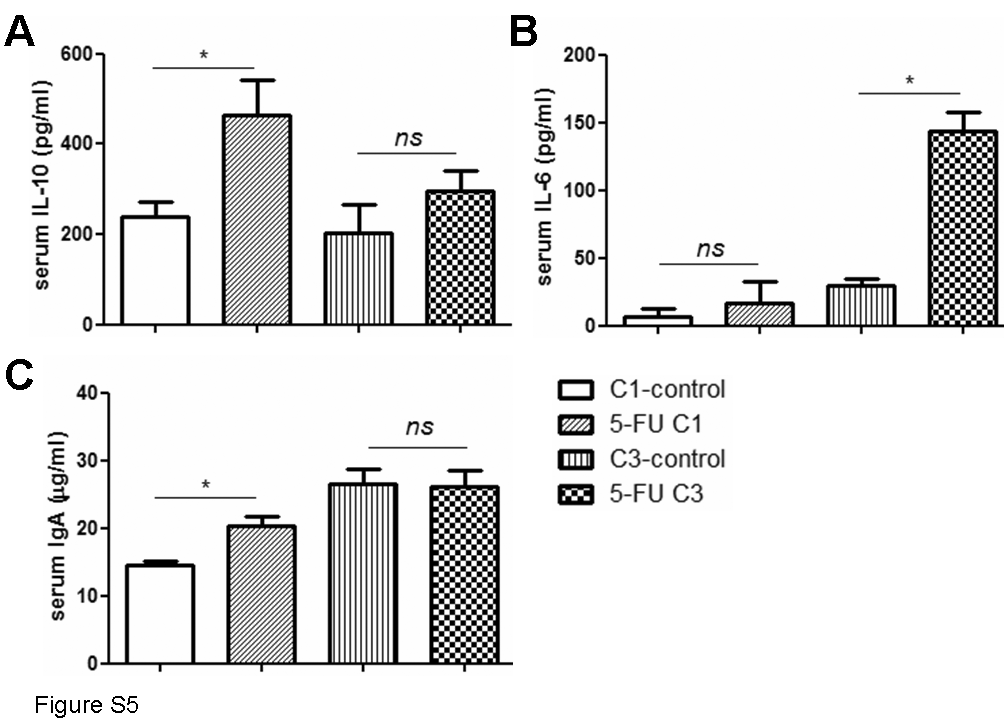

Supplement: Additional file 6: Figure S5. — (A) IL-10, (B) IL-6, and (C) IgA concentrations in serum of 5-FU treated and control mice. Serum was collected on day 7 after the last 5-FU injection, and cytokines were quantified by ELISA. Student’s t-test was used to analyze the significance between groups. The experiments were replicated at least twice with similar results. (TIF 943 kb) [file 12865_2016_167_MOESM6_ESM.tif]

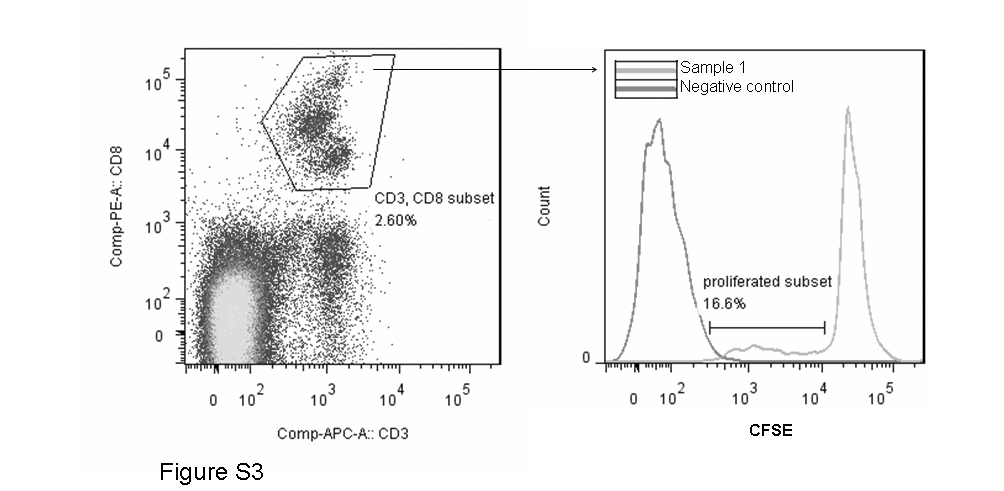

Supplement: Additional file 7: Figure S3. — Determination of proliferated CD8 T-cells by CFSE assay. CD8 T-cells were gated and calculated using their absolute number multiplied by their proliferated percentage (the cells with reduced CFSE expression were proliferated cells) to calculate the proliferated cell numbers. Proliferated CD4 T-cells, NK cells and B cells were detected in the same manner. (TIF 635 kb) [file 12865_2016_167_MOESM7_ESM.tif]

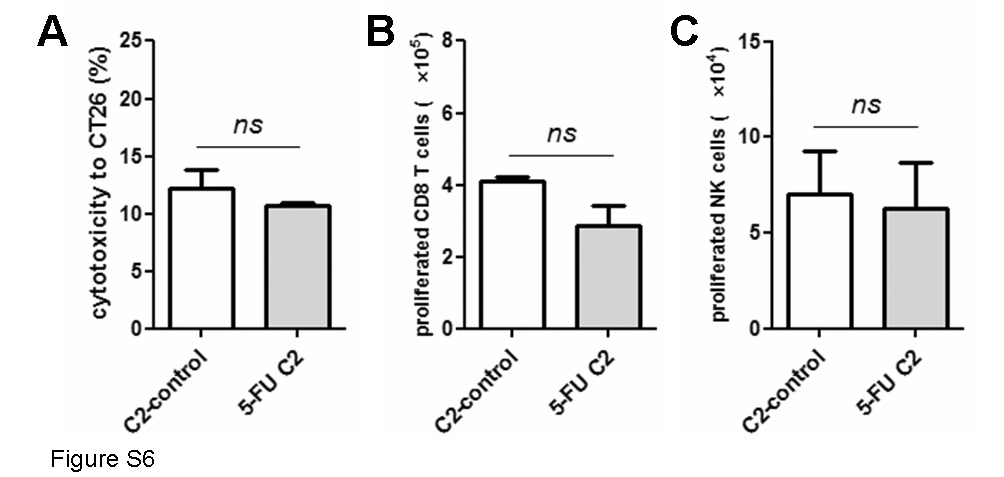

Supplement: Additional file 10: Figure S6. — In vitro immune functions of spleen cells against CT26 after 5-FU C2 treatment. (A) Cytotoxicity of spleen cells against CT26 from C2 and control groups were analyzed at the E:T ratio of 25:1. Proliferation of CD8 T cells (B) and NK cells (C) against CT26 were determined at the R:S ratio of 10:1 by CFSE assay. (TIF 618 kb) [file 12865_2016_167_MOESM10_ESM.tif]

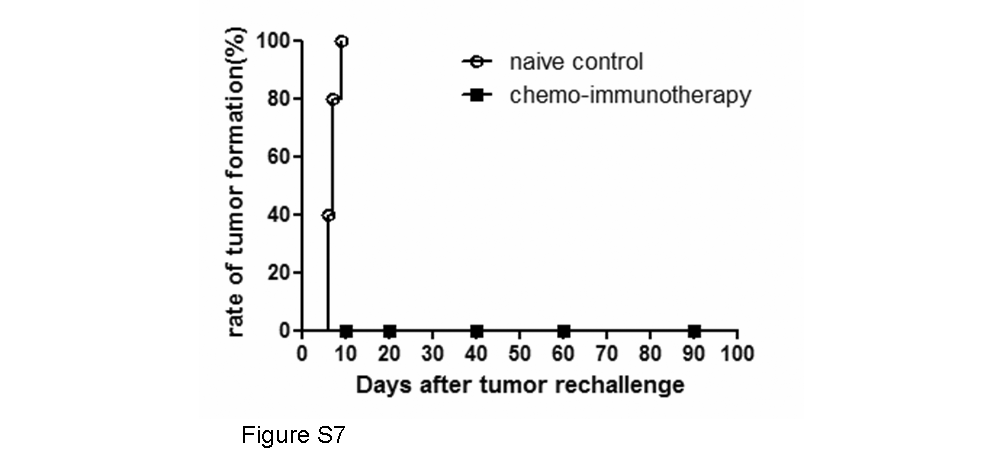

Supplement: Additional file 11: Figure S7. — Cured mice after chemo-immunotheapy (i.e., C1+ (CIK + α PD-L1)) were resistant to CT26 rechallenge. One month after final administration of CIKs and PD-L1 antibodies, cured mice and naïve control mice were inoculated with 1 × 10^6^ CT26 on the opposite side. Tumor formation were monitored and calculated. (TIF 541 kb) [file 12865_2016_167_MOESM11_ESM.tif]
